# Modeling Raccoon (*Procyon lotor*) Habitat Connectivity to Identify Potential Corridors for Rabies Spread

**DOI:** 10.3390/tropicalmed2030044

**Published:** 2017-08-28

**Authors:** Timothy P. Algeo, Dennis Slate, Rosemary M. Caron, Todd Atwood, Sergio Recuenco, Mark J. Ducey, Richard B. Chipman, Michael Palace

**Affiliations:** 1 USDA, APHIS, Wildlife Services, National Rabies Management Program, Concord, NH 03301, USA; dennis.slate@aphis.usda.gov (D.S.); richard.b.chipman@aphis.usda.gov (R.B.C.); 2 Department of Health Management and Policy, University of New Hampshire, Durham, NH 03824, USA; rosemary.caron@unh.edu; 3 USDA, APHIS, Wildlife Services, National Wildlife Research Center, Fort Collins, CO 80521, USA; tatwood@usgs.gov; 4 National Center for Public Health (Insitituto Nacional de Salud), Capac Yupanqui 1400, Jesus Maria, Lima 15073, Peru; srecuencoc@unmsm.edu.pe; 5 Department of Natural Resources and the Environment, University of New Hampshire, Durham, NH 03824, USA; mjducey@unh.edu; 6 Department of Earth Sciences, University of New Hampshire, Durham, NH 03824, USA; palace@guero.sr.unh.edu

**Keywords:** circuit theory, habitat suitability, Maxent, pine, *Pinus*, *Procyon lotor*, rabies, raccoon, risk model

## Abstract

The United States Department of Agriculture (USDA), Animal and Plant Health Inspection Service (APHIS), Wildlife Services National Rabies Management Program has conducted cooperative oral rabies vaccination (ORV) programs since 1997. Understanding the eco-epidemiology of raccoon (*Procyon lotor*) variant rabies (raccoon rabies) is critical to successful management. Pine (*Pinus* spp.)-dominated landscapes generally support low relative raccoon densities that may inhibit rabies spread. However, confounding landscape features, such as wetlands and human development, represent potentially elevated risk corridors for rabies spread, possibly imperiling enhanced rabies surveillance and ORV planning. Raccoon habitat suitability in pine-dominated landscapes in Massachusetts, Florida, and Alabama was modeled by the maximum entropy (Maxent) procedure using raccoon presence, and landscape and environmental data. Replicated (*n* = 100/state) bootstrapped Maxent models based on raccoon sampling locations from 2012–2014 indicated that soil type was the most influential variable in Alabama (permutation importance PI = 38.3), which, based on its relation to landcover type and resource distribution and abundance, was unsurprising. Precipitation (PI = 46.9) and temperature (PI = 52.1) were the most important variables in Massachusetts and Florida, but these possibly spurious results require further investigation. The Alabama Maxent probability surface map was ingested into Circuitscape for conductance visualizations of potential areas of habitat connectivity. Incorporating these and future results into raccoon rabies containment and elimination strategies could result in significant cost-savings for rabies management here and elsewhere.

## 1. Introduction

Rabies kills approximately 59,000 humans world-wide annually [1,2], but due to considerable control efforts, human cases are relatively rare in the developed world [1]. However, rabies control is expensive. Annual costs of living with rabies in the U.S. have been estimated at $300 million [3] ($646 million in 2017 USD [4]). Oral rabies vaccination (ORV) has proven effective for achieving wildlife rabies control [5] with noteworthy successes from a number of locations [6,7,8,9,10,11,12]. Raccoon (*Procyon lotor*) variant rabies (raccoon rabies) is currently present along the entire eastern seaboard, from Florida west to Alabama, north to the Canadian frontier, and west to Ohio. Consequently, it is one of the most important terrestrial variants currently circulating in North America in terms of incidence rates and proximity of raccoons to humans. Elimination of raccoon rabies is currently a high priority in the U.S. The United States Department of Agriculture (USDA), Animal and Plant Health Inspection Service (APHIS), Wildlife Services (WS) and cooperators have conducted ORV aimed at preventing the spread of raccoon rabies to the west (Phase I) since 1997. Phase II planning is underway and will focus on eliminating raccoon rabies from enzootic areas in the eastern U.S.

Considerable effort has been made to understand and model the spread, perpetuation, control, and economics of rabies. Deterministic and stochastic models for the spread of rabies in wildlife and for control scenarios with varying vaccination levels and barrier widths were evaluated, and the latter outperformed the former at rabies persistence and elimination simulations [13]. In Ontario, a time-series analysis was employed to try to understand regional differences in the dynamics of Arctic fox (*Alopex lagopus*) rabies in red foxes (*Vulpes vulpes*) [14], which ultimately led to the delineation of different rabies units with independent management strategies. Modeling has also demostrated geographic clustering of raccoon rabies cases in New York, and allowed for consideration of possible causes for these and related temporal patterns [15]. Additionally, modeled likely consequences of global climate change include a potential primary reservoir shift for Arctic fox variant rabies from Arctic to red foxes in Alaska [16], and range expansion for common vampire bats (*Desmodus rotundus*) [17]. Economic models of rabies and its control have been created for diverse scenarios, with the monetary burden from raccoon rabies estimated at $1.1 billion without ORV intervention over a 22-year horizon from 2012 to 2033 [18].

The concept of connectivity, or the ease of movement between landscape features, is important to understand in the contexts of both conservation and eco-epidemiology [19]. Connectivity modeling procedures are designed to identify critical movement areas for species of concern to achieve any of a number of management goals [20,21]. A relatively new approach to connectivity modeling is the adoption of circuit theory, in which the principles of electrical circuits are applied in ecology for creating more robust representations of and effects from known sources of resistance and multiple available pathways [22]. Through the incorporation of random walk theory [23], and Ohm′s Law-like resistances (wherein current = voltage/resistance, or I = V/R) in the form of resistance (or conductance) maps generated through habitat suitability index (HSI) modeling, circuit theory modeling can suggest pathways for the greatest likelihood of movement of members of a species between nodes or regions [22].

In the absence of complete information on habitat or site occupancy, assessments of distribution and habitat or site preference become problematic. The Maxent approach [20,24] allows for the use of occurrence data without absence information, along with environmental data, to estimate distribution likelihood for a given landscape. The Maxent procedure output includes a receiver operating characteristic (ROC), with the area under curve (AUC) provided as a measure of performance of the model in terms of assessing habitat suitability. Used in tandem with Circuitscape [25], a visualization of likely areas of concern for managers is created and in the case of rabies, can delineate priority areas for surveillance and control.

Comparisons between Maxent and other available procedures suggest at least equal performance and often superiority for Maxent in many cases. For example, Poor et al. [26] compared Maxent to the expert-based analytic hierarchy process (AHP) for developing habitat suitability models (HSM). They deemed the overall performance of the two HSM procedures to be similarly satisfactory, except that they considered Maxent′s cell-based habitat analyses inflexible compared with the expert-based approach in which temporal and spatial analysis units can be manipulated. However, they felt that Maxent performed somewhat better at ultimately producing more corridors that contained pronghorn antelope (*Antilocapra americana*) locations when the surfaces produced were utilized in connectivity models. A consideration worth noting is that in cases of relatively small sample sizes, information criteria procedures such as Akaike′s information criteria for small sample sizes (AICc) [27] or Bayesian information criteria (BIC) [28] provide somewhat better results than does the Maxent default AUC measure of model performance [29]. In another example, traditional movement models for golden-headed lion tamarins (*Leontopithecus chrysomelas*) in Brazil used Circuitscape [25] with an HSI resistance map to increase the authenticity of the modeled conservation situation in terms of tamarin movements between patches [30]. Circuitscape was also compared to least cost method (LCM) procedures for modeling connectivity using surfaces created through both the Maxent and AHP processes. While the corridors created using Maxent input generally contained more pronghorn antelope locations, the LCM proved superior in terms of generating pronghorn-containing corridors [26]. However, the ease with which Circuitscape′s seamless integration of Maxent outputs provides useful visualizations of landscape connectivity help it retain its appeal.

The WS ORV program along the Appalachian Ridge and the New England/New York-Canada frontier is designed to take advantage of research findings indicating relatively low raccoon densities at higher elevations (>610 m), as established by raccoon density indexing (RDI) [31], and which are presumed to result in reduced raccoon contact rates. Also of importance, the spread of raccoon rabies appears to be negatively affected by rivers [32,33,34] and certain types of forested habitats [33,34].

Much of the area targeted for Phase II is comprised of pine-dominated (*Pinus* spp.) forests. For example, pitch pine (*P. rigida*) occurs in coastal areas from New England south through New Jersey; Virginia pine (*P. virginiana*) occurs from the mid-Atlantic coast inland to the Appalachian Ridge-south; longleaf pine (*P. palustris*) is found in coastal areas from southern Virginia to Texas, and loblolly pine (*P. taeda*) is found in large commercial plantings in the southeast [35]. Relatively low RDIs have been developed for pitch pine and pitch pine-scrub oak (Q*uercus* spp.) forest types of southeastern Massachusetts and New Jersey [36]. Similarly, RDIs developed within loblolly and longleaf pine-dominated landscapes were lower relative to adjacent types [34,37].

We modeled raccoon habitat suitability in pine-dominated landscapes in Massachusetts, Florida, and Alabama by the maximum entropy (Maxent) procedure [20] using raccoon presence and landscape and environmental data to optimize ORV operations in pine-dominated landscapes of the eastern United States. As is the case for many generalist mesocarnivores, absence data for raccoons are difficult to acquire. Consequently, the presence-only Maxent procedure was employed given the relatively large number of locations available to us as byproducts of rabies management activities. Environmental surfaces generated by Maxent were ingested into Circuitscape for conductivity analysis [21,22,25,26,38], which then illustrated potential risk pathways for the spread of raccoon rabies. These results provide us an opportunity to assess the potential utility of circuit theory modeling for providing insight into critical areas for consideration when developing rabies control strategies.

## 2. Methods

Raccoon sampling location data are collected as part of routine rabies virus and post-ORV rabies serological monitoring which occurs in diverse habitats, including pine-dominated sites. Post-ORV live-trapping was conducted for the purpose of program assessment randomly throughout treated areas using Tomahawk Model 608 live-traps (Tomahawk Live Trap LLC, Hazelhurst, WI, USA). Raccoon handling was as described in Slate et al. [39]. Data from unique raccoons (*n* = 2986) sampled in Massachusetts, Florida, and Alabama during 5 January 2012–26 June 2014 were analyzed in Maxent. This sampling timeframe was selected to overlap with contemporary land use status as recorded within the National Land Cover Database 2011 (NLCD 2011) [40]. Trap location selection was based on opportunity (safe locations—for trappers, raccoons, and the public), where observed microhabitats suggested that potential undiscovered (by the public) raccoon capture was likely. While this does not reflect true random sampling, we made every effort to remove the influence of assumptions about density to achieve the greatest degree of randomness we could expect given the study limitations. Consequently, 1869 raccoons met study selection criteria (captured/collected by WS since 1 January 2012; not captured within 30 days prior; not captured as part of density indexing; and with a geo-location available), and their locations were imported into the GIS (Table 1).

Additional selection within the study areas in three states and GIS layer coverages resulted in 1770 raccoon sample locations available for Maxent model training and Circuitscape risk analysis.

### 2.1. Landscape Data

Land class and use types were represented by 30 m NLCD 2011 data [40] for the areas of interest. Although not the only available coverage for assessing forest cover, land use, and other surface feature types, NLCD 2011 is the standard used by WS for flight planning. Consequently, modeling results based on this product can be more easily translated into management actions. Study area characteristic analysis indicate that evergreen forest (NLCD 2011 Class 42) ranks 3/15 classes available for the Massachusetts study area, 9/13 for the Florida study area, and 4/15 in Alabama. NLCD 2011 Class representation among raccoon sample locations used in initial analyses indicated that 8.8% of raccoon sample locations in Massachusetts fell into the evergreen class, while only 5.3% and 6.2% were in that class in Florida and Alabama, respectively (Table 2).

The Digital General Soil Map of the U.S. (STATSGO2) represents an inventory of soils mapped at 1:250,000 scale [42] and at the level of soil taxonomic order that provides appropriate resolution data for consideration at raccoon home range scales. Study area characteristic analysis in terms taxonomic soil orders (soils) indicates that entisols (dunes, floodplains) rank highest among soil orders for the Massachusetts study area (>98%), while in the Florida and Alabama study areas, soils from the alfisols (common in semiarid-moist regions) dominated at >67% and almost 42%, respectively. In terms of soils representation among the sample points used in initial analyses, >99% (*n* = 171) of raccoon sample locations in southeastern Massachusetts fell into the entisols, while in Florida the majority (almost 88%) fell into the alfisols. A greater diversity of soils was represented among the Alabama raccoon sample locations (alfisols at 34%, entisols at 37%, inceptisols (characterized by varied productivity; layer formation developing) at 19%, and ultisols at 9%).

### 2.2. Environmental Data

Euclidean distance layers for distance to National Hydrography Dataset (1:24,000) lake, pond, swamp, marshland, reservoir, and estuary (nhd24kwb; water bodies); and stream, river, canal, ditch and coastline (nhd24kst; streams) data [43] were also incorporated. Environmental inputs were 1 km Worldclim Bio1 (Annual Mean Temperature; temperature) and Bio12 (Annual Mean Precipitation; precipitation) data [44]. Worldclim elevation (elevation) data [44] derived from the Shuttle Radar Topography Mission (SRTM) [45] were also used for assessing potential effects from elevation to RDI (Table 3).

### 2.3. Human Environment Data

To refine our assessment of potential effects from human development that may subsidize raccoon populations through the provision of garbage, garden and farm crops beyond what is demonstrated in the development classes within the NLCD 2011 [40], 2010 census data in the form of population and housing layers were incorporated [46]. Euclidean distance to primary and secondary road layers [46] for our study areas were also included, given the potential role of roads as travel corridors or barriers to movement (Table 3).

### 2.4. Data Preparation and Processing

Circuitscape requires ASCII raster-formatted data for analysis. The Circuitscape Exporter add-in tool for ArcMap [47] was used to export ArcGIS vector and raster data into ASCII rasters of identical cell sizes, extents, and spatial references for use in Maxent, and for eventual ingestion into Circuitscape. Separate global Maxent model (settings: 100 replicate bootstrap analysis, 25% random-seeded test percentage, logistic output format, duplicate locations allowed, response curves created, jackknife measure of variable importance, and 5000 maximum iterations) runs were made for each state using raccoon sample locations from 2012-present and 10 ‘environmental layers’ (Table 3).

Maxent created ROC plots of true versus false positive locations in the model, and an assessment of the AUC created by plotting sensitivity (1-omission rate) against 1-specificity (the fractional predicted area), which is a threshold-independent measure of accuracy and ranges from between 0.5 (the random prediction) to the maximum achievable value of 1.0 [49]. Percent contribution and permutation importance values provided measures of variable performance, with the former being useful for assessing the roles of uncorrelated variables, and the latter for an assessment based on the AUC metric of the dependence of the model on a given variable. The jackknife results provide a visual interpretation of variable importance [50]. Resulting environmental surfaces were ingested into Circuitscape for conductivity analysis [25,38].

In Circuitscape, the input resistance data (environmental data) were set to represent conductances rather than resistances, and the focal nodes (samples) consisted of 500 random points created in ArcMap for the global models. To expedite analyses, the all-to-one modeling mode was selected. Resulting Circuitscape conductance maps provide no metrics, but despite this are extremely useful for visualizing areas of concern for the prevention of the spread, or the perpetuation, of rabies in our areas of interest. 

In addition, for each environmental layer class (environmental = temperature and precipitation; landscape = NLCD 2011, elevation, stream, water body, and soils; and human-based = human population, housing, and roads) separate models were constructed to further develop an understanding of the importance of these variables as classes of landscape factors. A 5-replicate bootstrap Maxent analysis (all other settings as above) was conducted for each of these models. Circuitscape images were not created for these analyses.

## 3. Results

Raccoon sample location distribution (*n* = 171) was relatively even across areas of interest in Barnstable and Plymouth Counties, Massachusetts. The averaged 100-replicate bootstrapped Maxent model ROC AUC (model sensitivity relative to 1-specificity) for southeastern Massachusetts was relatively high at 0.958. Higher levels of raccoon sample location probability are seen along the more heavily populated (by humans) and mixed-forest type dominated coasts. Precipitation was the most influential variable in the Massachusetts Maxent model (44% contribution; 46.9 permutation importance), with a higher proportion of raccoon sample locations occurring where precipitation levels were higher, primarily along the outer portion of Cape Cod. The next most influential variable was NLCD 2011 (15.3%; 7) which had more raccoon sample locations (40.9%) occurring on the developed low intensity (NLCD 2011 Class 22) than any other land class available, while soils was the third most influential (11.7%; 3.8). All other variables fell below 10% model contributions, although permutation importance was higher for several of these than for the second and third highest in terms of percent contributions (Table 4).

The Maxent model for raccoon sample (*n* = 431) distribution in Florida performed slightly better than the model for Massachusetts, with an AUC >0.975. The distribution of raccoon sample locations and the resulting Maxent model representation for Florida demonstrate a potential difference between several urbanized areas in terms of predicted raccoon habitat suitability. Temperature played the most important role in raccoon sample collection location in Florida (30.1%, 52.1), with the majority of raccoons at locations with slightly higher annual mean temperatures. Precipitation played the next most important role (21.5%, 10.5). Soils was the third most important (20.5%, 19.4) variable. All other variables made <10 percent contributions and scored below the topmost variables in terms of permutation importance as well (Table 5).

Of the three states sampled, the Maxent model for raccoon sample (*n* = 1168) location distribution in Alabama performed the least well, with an AUC of 0.932. Here, soil was the most influential variable (39.4%, 38.3), followed by temperature (18.3%, 22.7) and elevation (12.1%, 15.9) (Table 6).

**Figure 1:**
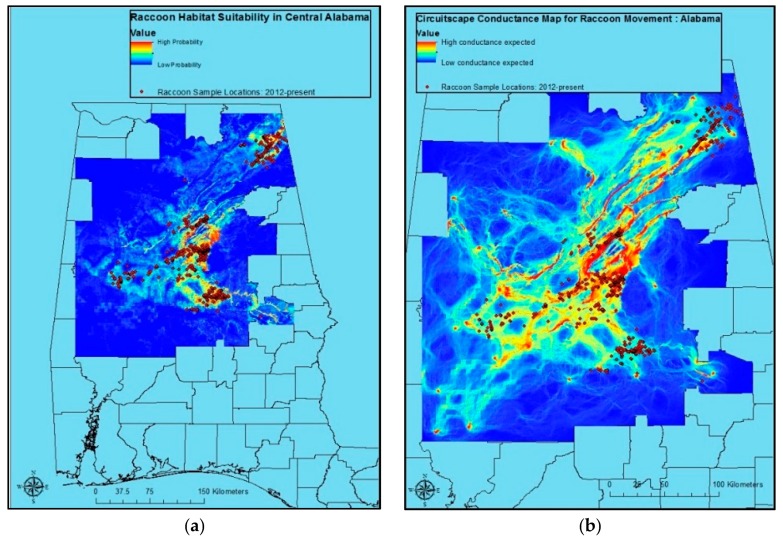
Maxent raccoon location probability map based on raccoon sample ((**a**); *n* = 1168; 5 January 2012–26 June 2014) locations, annual mean precipitation, annual mean temperature, elevation, land cover/land use, human population density, housing density, Euclidean distance to roads, USDA Soil Taxonomic Order, Euclidean distance to streams/rivers, and Euclidean distance to water bodies for central Alabama; and Circuitscape conductance map for raccoon rabies risk in Alabama (**b**).

Raccoon sample locations and predicted raccoon habitat suitability for Alabama were fairly consistent. However, areas with relatively high probability predictions without raccoon samples having come from them were evident as well (Figure 1a). The Circuitscape output based on Maxent results for Alabama reveals areas of considerable risk for the movement of raccoon rabies (Figure 1b).

**Figure 2:**
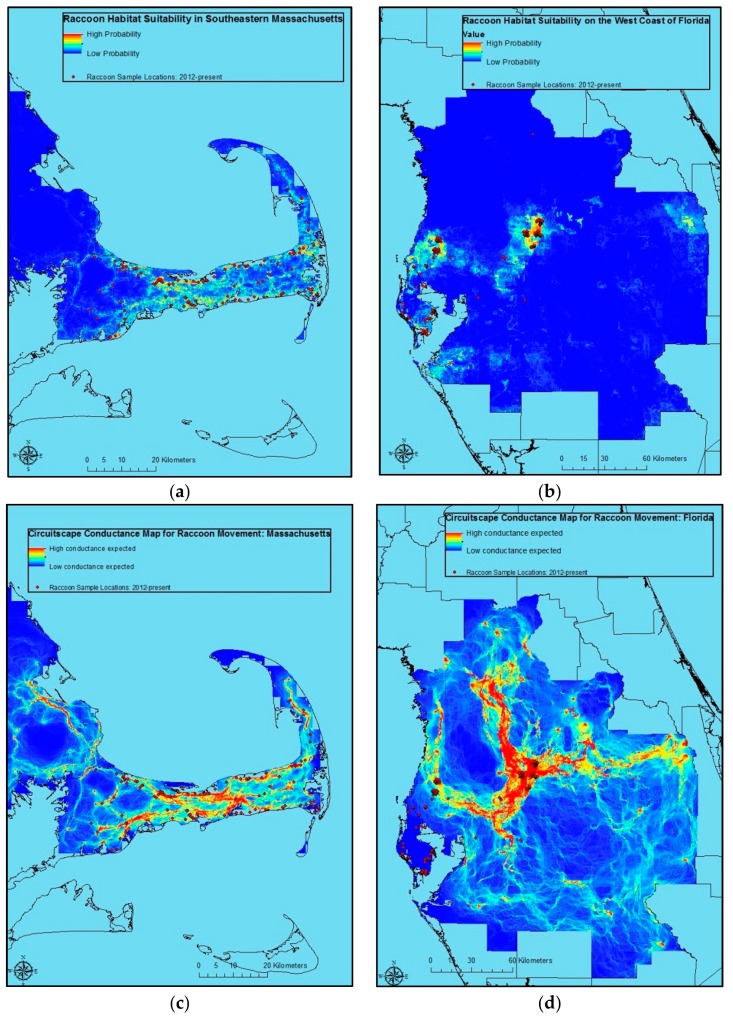
Maxent raccoon location probability map based on samples from Massachusetts(**a**; *n* = 171) and Florida (**b**; *n* = 431) collected during 5 January 2012–26 June 2014) plus annual mean precipitation, annual mean temperature, elevation, land cover/land use, human population density, housing density, Euclidean distance to roads, USDA Soil Taxonomic Order, Euclidean distance to streams/rivers, and Euclidean distance to water bodies; and related Circuitscape conductance maps for raccoon rabies risk in Massachusetts (**c**) and Florida (**d**).

Raccoon sample locations and predicted raccoon habitat suitability were also relatively consistent in Massachusetts and Florida (Figure 2a,b). As in Alabama, Circuitscape predicts raccoon movement risk (Figure 2c,d). However, given concerns over model results relative to program understanding of raccoon ecology, these are potentially less useful for management decision-making than the model for Alabama (Figure 1b).

Evaluated as groupings of like variables, the landscape suite performed slightly better than the environmental and human-based ones in terms of average AUC, with relatively high mean AUC values in both Florida and Alabama. Only Florida had a mean AUC that exceeded 0.90 for the environmental suite of variables, while none in the human-based category did (Table 7).

## 4. Discussion

The Maxent procedure provided unexpected results suggesting relatively strong environmental influence on raccoon distribution probability for both Massachusetts (precipitation) and Florida (temperature), while the contribution of soil in each was less important than would have been expected, given its relationship to landcover. In Alabama, the high percent contribution of soils data was less surprising given the influence of soils on not only the distribution of forest types, but also on water retention and very likely the distribution of invertebrate prey items. The strong influence of environmental variables suggests further investigation is warranted for Massachusetts and Florida. The three additional Maxent models run to explore the relative influence of groupings of variables in the absence of other variable types revealed a somewhat higher level of influence for the landscape class in terms of their average AUC, which is more in line with our expectations given what is known about raccoon ecology.

No variable or variable class ultimately emerged as the best all-around predictor in the settings modeled. However, the most influential variables in each state had strong effects in comparison to second tier variables. For example, in Massachusetts, precipitation with a percent contribution of 44 was also extremely influential when evaluated by itself, with the highest level of permutation importance (46.9) (Table 4). For Florida and Alabama, differences between percent contributions of the best performing variables were less dramatic.

Also in Massachusetts, the NLCD 2011 variable (15.3%; 7) was expected to be highly influential. However, it performed only slightly better than soils (11.7%; 3.8), whereas soils greatly outperformed NLCD 2011 in Florida and Alabama. Results from the soils indicated that almost all (>99%) raccoon samples came from within the entisols in Massachusetts, which characterize disturbed areas and which may be explained by relatively high levels of development and attendant soil disruption. In Florida, the alfisols dominated with almost 88% of samples originating there. Not surprisingly, these soils are somewhat characteristic of humid locations. In contrast, raccoon sample locations in Alabama were distributed among a number of soil types, with alfisols and entisols being almost equally represented among raccoon samples there at 34.4% and 36.6%, respectively. One exception was the inceptisols, which while they represented >25% of the Alabama study area, accounted for only 19.1% of raccoon sample locations.

Although neither human population nor housing ever represented >5.8% contribution toward influencing of raccoon sample locations, planning for ORV bait distribution within residential and recreational areas has always been a priority given our knowledge of raccoon use of household wastes, garden crops, and invertebrates found in lawns, etc. However, based on our results, it appears that bypassing relatively small residential areas when conducting large scale ORV operations may have little impact on overall program success. Further confirmation of this from directed density and movement assessments could lead to greater understanding and considerable cost-savings given the complications of baiting in areas such as these. Roads was never among the top-performing variables, with a high of only 11 percent influence in Alabama. This suggests raccoons may not be dependent on them as travel corridors, nor deterred by them as potential obstacles. A potential bias from road kill- and roadside trapping-based sampling among surveillance and trapped raccoons, may have confounded this finding, however.

The consistently poor performance of streams, water bodies, human population and housing variables was somewhat surprising given the frequently documented attractiveness of these features to raccoons [48,51]. While it seems unlikely, the apparent scarcity of resources available to raccoons in pine-dominated landscapes may also extend to riparian areas contained within them, possibly explaining the phenomenon. It is also possible that raccoon population densities are low enough in some of the pine-dominated landscapes sampled that even these localized resources are not abundant enough to attract and sustain them.

The relatively high level of influence from temperature in all three states is somewhat surprising as well (Table 4, Table 5 and Table 6). Although these findings suggest further consideration of the potential role of temperature in predicting raccoon sample locations, it might also be important to consider revising this analysis to incorporate higher-resolution temperature and precipitation data to better determine if there is a real measurable influence.

This modeling effort presented a number of challenges. For example, the raccoon samples utilized were byproducts of other research and management efforts. Much of the work done in these areas, and in particular in Massachusetts, was undertaken as emergency response to raccoon rabies epizootic front movements, barrier breaches, and perceived threats based on surveillance. In addition, samples were frequently road kills, and from residential areas where concerned citizens reported them. Finally, our primary knowledge of raccoon densities in these areas comes from 10-day, 500 trap night (with some allowance for initial results–based modifications), 3-km^2^ density estimation procedures designed to provide enough information for responsive ORV and conducted on the fly and with limited resources [31]. More refined findings may also be required for full understanding of the role of commercial pine plantations in the south. For instance, seral stage was an important predictor for determining raccoon usage of commercial loblolly pine plantation habitat, and raccoon home ranges overlapped more frequently in commercial pine forests than in mixed forest types in one study in Alabama [34]. Consequently, further analysis incorporating remotely-sensed imagery capable of elucidating seral stage in near real time may be necessary for full understanding on commercial pine plantation-dominated landscapes. In addition, since edge habitats have emerged as important predictors of relative raccoon density in pine-dominated forest types [36], incorporating this landscape feature into future Maxent assessments may prove useful.

Applying the Maxent and Circuitscape procedures to problems such as we addressed here was useful. However, the application of these findings to future management efforts will occur only after a process that includes other inputs. Our examination of variable permutation importance aids in discerning variable input on model performance. However, for similar future analyses, we feel additional evaluation in the form of correlation analysis will provide an even clearer understanding of the potential problem of collinearity. Nonetheless, these Circuitscape outputs make clear to us areas for strong consideration for sampling in rabies surveillance efforts, and for concentrating treatment by ORV, or in limited cases, by trap-vaccinate-release (TVR). Although many of the higher risk corridors suggested jibe with what we know about raccoon rabies eco-epidemiology in these regions, we are aware of the limitations of modeling for management, regardless of the procedures utilized. The NRMP has traditionally modified management strategies based on modeling only after careful consideration of outputs in light of what is known from actual work on the ground, and then only where over-arching programmatic risks can be minimized.

## 5. Summary

Relatively new modeling tools such as the maximum entropy procedure (Maxent) [20,25] hold promise for use in the field of eco-epidemiology by helping managers increase their understanding of the systems in which they work to control important diseases such as rabies. Although Circuitscape conductance maps provide no metrics, they are extremely useful for visualizing areas of concern for the prevention of the spread or the perpetuation of rabies in areas of interest. As such, they actually represent the input of greatest interest to discussions of management options and strategy formulation. If ORV or trap-vaccinate-release operations are to be implemented, these will help direct those efforts geographically in much the same way that forest fire managers strategize to achieve control. In addition, potentially wasteful efforts in low conductance areas may be avoided, adding cost-savings.

No variable or variable class emerged as the best all-around predictor for the three locations under consideration. However, the Maxent procedure for Alabama provided us with useful results suggesting optimal raccoon habitat (Table 6, Figure 1a), as well as conductance surfaces for use in visualizing risk corridors potentially useful for planning ORV and contingency actions in response to ORV zone breaches in Circuitscape (Figure 1b). These results are consistent with anecdotal information from a number of years and sources on areas of optimal raccoon habitat, and highest raccoon rabies spread in that state. The resulting NLCD 2011 data were somewhat less informative in modeling raccoon habitat suitability than soils, except in Massachusetts where NLCD 2011 performed slightly better. However, the ease with which NLCD 2011-based research and planning are translated to field decisions make it unlikely that a switch to soil data in decision-making, research, etc. would occur. Further exploration of the potential ties between these types of information, and the possible development of algorithms for incorporating soils information into field decisions may be worth exploring, however.

Functionally equivalent forest types dominated by other members of *Pinus* occur along coastal portions of the Atlantic and Gulf of Mexico coasts as well, so the apparent pine effect noted here may be widespread within Phase II ORV treatment areas. Pine-dominated forests comprise significant proportions of the forest types of the eastern U.S. For example, 69 million acres of the southeastern U.S. is coniferous forest, with the majority of the area occupied by loblolly and longleaf pine [52], and pitch pine forests occur from as far north as southern Maine to northern Georgia, with concentrations found on the Atlantic coastal plain [35]. Considerable savings may accrue to ORV efforts dedicated to controlling raccoon rabies in pine-dominated regions by application of these and future findings. This effort may also suggest procedures of potential utility for controlling rabies elsewhere with different reservoirs species, and may provide important inputs for future economic modeling of rabies control.

## Figures and Tables

**Table 1:** Raccoon risk model sample data for pine-dominated landscapes of Massachusetts, Florida, and Alabama: 2012–2014. ^1^

State	Dates Sampled	Sample Size	Age Ratio ^2,3^ (n)	Sex Ratio (M:F; n)
Massachusetts	25 January 2012–26 June 2014	171	4.3:1 (32)	1.7:1 (168)
Florida	19 January 2012–30 May 2014	431	15.4:1 (278)	1.7:1 (427)
Alabama	5 January 2012–20 December 2013	1267	NA	1.2:1 (1251)
Total		1869	12.5:1 (310)	1.3:1 (1846)

^1^ Selection criteria for study inclusion: captured/collected by USDA, APHIS, Wildlife Services personnel since 1 January 2012; no capture within 30 days prior; not captured as part of a density study; geo-location available. Not all samples selected were ultimately included in each analysis. ^2^ Age ratios are adult: juvenile; juvenile status ≤1 year as aged by the *cementum annuli* procedure, Matson′s Laboratory, Milltown, MT [41]. ^3^ Age data not available for Alabama.

**Table 2:** NLCD 2011 ^1^ class representation in raccoon risk model study area and sample point data (5 January 2012–26 June 2014) from within pine-dominated landscapes of Massachusetts, Florida, and Alabama.

	Massachusetts (*n* = 171)		Florida(*n* = 431)		Alabama(*n* = 1168)	
NLCD 2011 Class ^1^	Percent Study Area	Percent Sample Points	Percent Study Area	Percent Sample Points	Percent Study Area	Percent Sample Points
11-open water	2.1	NA	1.5	NA	0.9	2.1
21-developed, open space	19.6	19.3	19.0	15.8	10.3	23.7
22-developed, low intensity	17.7	40.9	13.3	7.7	2.3	4.5
23-developed, medium intensity	8.7	6.4	6.1	1.6	0.8	0.9
24-developed, high intensity	1.2	0.6	1.4	0.9	0.2	0.3
31-barren land (rock/sand/clay)	2.0	NA	1.0	NA	0.4	0.7
41-deciduous forest	11.0	4.1	NA	NA	17.0	24.7
42-evergreen forest	11.7	8.8	3.7	5.3	12.8	6.2
43 mixed forest	8.5	1.2	NA	NA	20.4	5.2
52-shrub/scrub	2.7	NA	7.3	6.5	15.0	6.2
71-grassland/herbaceous	2.9	1.2	7.7	1.9	5.3	4.8
81-pasture/hay	1.1	NA	5.6	1.6	6.2	13.2
82-cultivated crops	0.7	NA	0.9	NA	2.4	3.2
90-woody wetlands	5.6	8.2	19.9	54.1	5.1	4.3
95-emergent herbaceous wetlands	4.6	9.4	12.6	4.6	0.6	0

^1^ Source: U.S. Geological Survey, 2014 [40].

**Table 3:** Raccoon risk modeling: environmental variables, sources, and choice justification for pine-dominated landscapes of Massachusetts, Florida, and Alabama.

Variable	Data Type	Source	Selection Justification
National LandcoverDataset (NLCD 2011)	Landscape/categorical	30 m GeoTIFF images-http://landcover.usgs.gov/	Used by NRMP for ORV planning
Euclidean Distance to (streams)	Landscape/continuous	National Hydrography Dataset 2014 http://nhd.usgs.gov/	Raccoon foraging frequently focused in riparian areas [48]
Euclidean Distance to water (water bodies)	Landscape/continuous	National Hydrography Dataset 2014 http://nhd.usgs.gov/	Raccoon foraging frequently focused in riparian areas [48]
USDA soil taxonomic order (soils)	Landscape/categorical	STATSGO22 http://www.nrcs.usda.gov/	Standing water, and invertebrate availability [48]
Elevation (elevation)	Landscape/continuous	Shuttle Radar Topography Mission http://www2.jpl.nasa.gov/srtm/	Likely correlated with water/NLCD
Annual Mean Precipitation (precipitation)	Environmental/continuous	WorldClim, Global Climate Data http://www.worldclim.org/	Standing water, land cover/use types
Annual Mean Temperature (temperature)	Environmental/continuous	WorldClim, Global Climate Data http://www.worldclim.org/	Foraging behavior and reproduction timing
Human Housing Density (housing)	Human environment/continuous	2010 Census Population/Housing Unit Counts-Blocks: Tiger/line files www.census.gov	Human subsidies to raccoons - garbage, garden crops [48]
Human Population Density (human population)	Human environment/continuous	2010 Census Population/Housing Unit Counts-Blocks: Tiger/line files www.census.gov	Human subsidies to raccoons - garbage, garden crops [48]
Euclidean Distance to Roads (roads)	Human environment/continuous	U.S. Census Bureau http://www.census.gov/geo/maps-data/data/tiger.html	Travel corridors

**Table 4:** Raccoon (*n* = 171) risk modeling sample data for pine-dominated landscapes of Massachusetts during 5 January 2012–26 June 2014. All values are means.

Variable	Percent Contribution	Permutation Importance
Annual Mean Precipitation	44	46.9
National Landcover Dataset 2011	15.3	7
USDA Soil Taxonomic Order	11.7	3.8
Euclidean Distance to Roads	9.5	9.1
Human Population Density	5.8	11.4
Human Housing Density	4.5	7
Annual Mean Temperature	3.6	4.8
Euclidean Distance to Streams	2.4	2.6
SRTM Elevation	2.3	5.9
Euclidean Distance to Water bodies	1	1.4

**Table 5:** Raccoon (*n* = 431) risk modeling sample data for pine-dominated landscapes of Florida during 5 January 2012–26 June 2014. All values are means.

Variable	Percent Contribution	Permutation Importance
Annual Mean Temperature	30.1	52.1
Annual Mean Precipitation	21.5	10.5
USDA Soil Taxonomic Order	20.5	19.4
Euclidean Distance to Roads	7.4	2.6
SRTM Elevation Data	7.1	6.6
Euclidean Distance to Water bodies	5.1	3
National Landcover Dataset 2011	4.6	3.1
Human Housing Density	2.9	1.8
Human Population Density	0.6	0.4
Euclidean Distance to Streams	0.1	0.5

**Table 6:** Raccoon (*n* = 1168) risk modeling sample data for pine-dominated landscapes of Alabama during 5 January 2012–26 June 2014. All values are means.

Variable	Percent Contribution	Permutation Importance
USDA Soil Taxonomic Order	39.4	38.3
Annual Mean Temperature	18.3	22.7
SRTM Elevation Data	12.1	15.9
Euclidean Distance to Roads	11	7.1
National Landcover Dataset 2011	8.3	2.4
Annual Mean Precipitation	6.1	11
Euclidean Distance to Water bodies	3.1	0.8
Human Population Density	1	1.2
Human Housing Density	0.6	0.4
Euclidean Distance to Streams	0.1	0.2

**Table 7:** Evaluation of environmental layer classes for their influence on raccoon risk model results for pine-dominated landscapes of Massachusetts, Florida, and Alabama during 5 January 2012–26 June 2014: 5 bootstrap replicated runs. All AUC values are means.

State	Model Performance	Environmental Model ^1^	Landscape Model ^2^	Human-based Model ^3^
MA	Mean AUC	0.886	0.872	0.861
	95% CI ±	0.0023	0.0024	0.0031
	n	166	153	171
FL	Mean AUC	0.956	0.963	0.824
	95% CI ±	0.0002	0.0004	0.0008
	n	411	396	431
AL	Mean AUC	0.858	0.916	0.749
	95% CI ±	0.0003	0.0001	0.0002
	n	1168	1168	1168

^1^ annual mean temperature and precipitation at the 1 km resolution; ^2^ NLCD 2011, elevation, Euclidean distance to streams, Euclidean distance to water bodies, and soils; ^3^ human population, human housing, and Euclidean distance to roads.
